# Preliminary experience using milnacipran in patients with juvenile fibromyalgia: lessons from a clinical trial program

**DOI:** 10.1186/s12969-015-0025-9

**Published:** 2015-06-26

**Authors:** Lesley M. Arnold, Lucinda Bateman, Robert H. Palmer, Yuhua Lin

**Affiliations:** Women’s Health Research Program, University of Cincinnati College of Medicine, 260 Stetson Street, Suite 3200, Cincinnati, OH 45219 USA; Fatigue Consultation Clinic, 1002 E. South Temple Street, Suite 408, Salt Lake City, UT 84102 USA; Forest Research Institute, an affiliate of Actavis, Inc., Harborside Financial Center, Plaza V, Suite 1900, Jersey City, NJ 07311 USA

**Keywords:** Juvenile fibromyalgia, Milnacipran, Pain, Quality of life

## Abstract

**Background:**

There are no approved medications for juvenile fibromyalgia (JFM), a disorder that is often under-diagnosed. The effects of milnacipran, a drug approved for the management of fibromyalgia (FM) in adults, was assessed in a clinical trial program for JFM.

**Methods:**

Patients, ages 13–17 years who met the Yunus and Masi criteria for JFM and/or 1990 American College of Rheumatology criteria for FM, were enrolled in a responder-enriched, randomized withdrawal trial. After receiving open-label milnacipran (8 weeks), patients with ≥50 % improvement in pain underwent double-blind randomization (1:2) to either placebo or continuing treatment with milnacipran (8 weeks). All patients, including those who did not meet the randomization criteria for double-blind withdrawal, were allowed to enter an extension study with open-label milnacipran (up to 52 weeks). The primary endpoint was loss of therapeutic response (LTR) during the double-blind period. Additional outcome measures included the Patient Global Impression of Severity (PGIS), Pediatric Quality of Life Inventory (PedsQL: Generic Core Scales, Multidimensional Fatigue Scale), and Multidimensional Anxiety Scale for Children (MASC). Safety assessments included adverse events (AEs), vital signs, electrocardiograms, and laboratory tests.

**Results:**

The milnacipran program was terminated early due to low enrollment. Because only 20 patients were randomized into the double-blind withdrawal period, statistical analyses were not conducted for the LTR endpoint. However, 116 patients entered the open-label period of the initial study and 57 participated in the open-label extension study. Their experience provides preliminary information about the use of milnacipran in JFM patients. During both open-label periods, there were mean improvements in pain severity, PGIC, PedsQL, and MASC scores. No unexpected safety issues were detected. The most commonly reported treatment-emergent AEs were nausea, headache, vomiting, and dizziness. Mean increases in heart rate and blood pressure were observed, and were consistent with the AE profile in adults with FM.

**Conclusions:**

The open-label findings provide preliminary evidence that milnacipran may improve symptoms of JFM, with a safety and tolerability profile that is consistent with the experience in adult FM patients. Future trial designs for JFM should consider the relatively low recognition of this condition compared to adult FM and the difficulties with enrollment.

**Trial registration:**

NCT01328002; NCT01331109

**Electronic supplementary material:**

The online version of this article (doi:10.1186/s12969-015-0025-9) contains supplementary material, which is available to authorized users.

## Background

Juvenile fibromyalgia (JFM) is a chronic pain disorder that affects an estimated 1.2 % to 6.2 % of children and adolescents in the general population, more frequently in girls than boys [1–3]. JFM accounts for an estimated 7 % to 15 % of referrals to pediatric rheumatology clinics [4] and has been found in 52 % of female adolescents who were admitted for inpatient psychiatric treatment [5]. Recognizing this disorder can be challenging, and patients may consult with several different clinicians before receiving a diagnosis of JFM [4]. JFM can be identified based on a number of characteristic symptoms, including diffuse and chronic musculoskeletal pain, fatigue, sleep difficulties, headaches, anxiety, and depressed mood [2]. In clinical studies, diagnosis is usually based on the Yunus and Masi criteria for JFM [6] or the 1990 American College of Rheumatology (ACR) fibromyalgia (FM) criteria for adults [7].

As with adult FM, it has been hypothesized that JFM involves abnormal pain processing in the central nervous system, which results in hypersensitivity to painful and nonpainful stimuli [4]. The symptoms and prognosis for the disorder may also be affected by factors such as family and peer relationships, emotional and social functioning, and the presence of anxiety or mood disorders (lifetime or current) [8–10]. Given the multisymptomatic nature of JFM, along with the complex neurobiological and psychosocial components, an individualized and multidisciplinary treatment approach is generally recommended [2]. Cognitive behavioral therapy (CBT) may help patients change their perceptions about pain and develop coping skills to navigate the challenges of JFM [11]. In adolescents with JFM, this therapeutic approach has been shown to improve pain coping and functional ability [12–16]. Exercise programs may also provide therapeutic benefits, such as increased physical activity and improved quality of life [17, 18].

No medications have been approved for the management of JFM, although results from a preliminary trial of fluoxetine in JFM have been published [19]. Three medications are currently approved for the management of FM in adults: an alpha-2-delta ligand, pregabalin, which may modulate pain by decreasing neurotransmission of glutamate and substance P; and 2 serotonin and norepinephrine reuptake inhibitors, duloxetine and milnacipran, which may target the descending central neural pathways involved in pain inhibition [20]. In placebo-controlled trials with adult FM patients, all 3 medications demonstrated statistically significant improvements in pain, function, and global assessment of improvement. Pregabalin, duloxetine, and milnacipran have also been shown to have positive effects on secondary symptom domains of FM. For example, duloxetine and milnacipran were shown to be effective for fatigue and depressive symptoms [21–23], milnacipran for self-reported cognitive difficulties [24, 25], and pregabalin for sleep disturbances [26, 27]. These various effects in adult FM patients are consistent with pharmacologic differences among the medications, including the greater effect of milnacipran on norepinephrine reuptake relative to duloxetine [28]. These differences are also reflected in the side-effect profiles of the medications [29–31]. Patients with FM are a heterogeneous group and multiple treatment options are important to address the variability of symptoms and impact of FM [32].

A clinical trial of pregabalin in JFM was recently completed (NCT01020474), the results of which are currently being analyzed and are as yet unpublished [33]; a JFM study with duloxetine is ongoing (NCT01237587). The JFM clinical trial program with milnacipran consisted of a responder-enriched, randomized withdrawal study (NCT01328002 [MLN-MD-14]) followed by an open-label extension study (NCT01331109 [MLN-MD-29]). This program, described in the current report, was terminated early because of difficulties recruiting an adequate number of patients who had been diagnosed with JFM and met the study criteria. However, demographic data obtained on a comparatively large group of patients with JFM (*n* = 116), along with safety and preliminary findings on outcome measures, may provide important contemporary characterizations of these patients and be helpful in designing future JFM trials.

## Methods

### Program overview

**Fig. 1:**
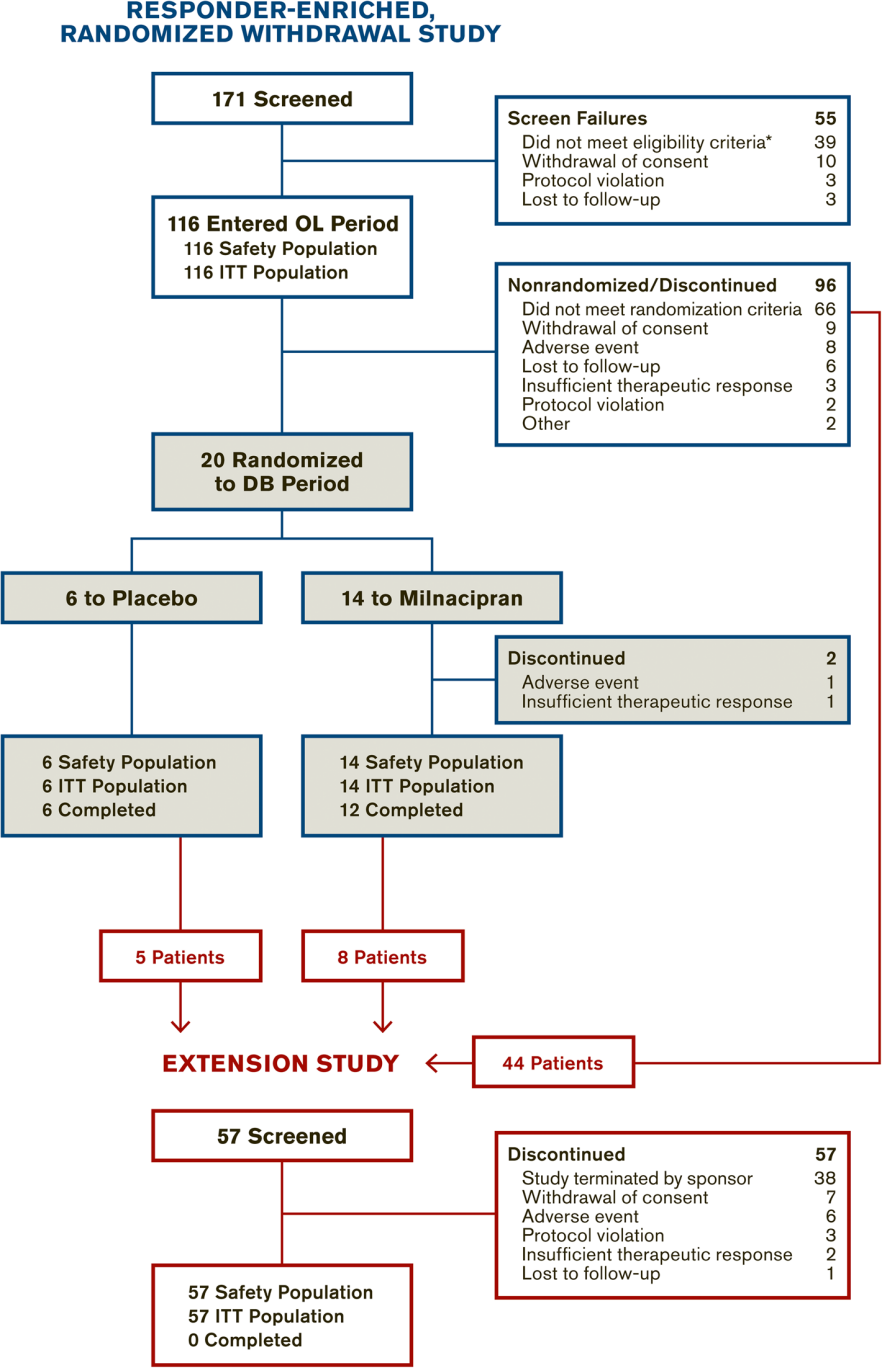
Study flow. *Reasons for ineligibility occurring in >1 patient: current severe psychiatric illness (12 patients); did not have a mean daily pain rating of ≥3 to ≤ 9 in the week prior to the baseline visit (8 patients); did not meet Yunus and Masi criteria or ACR criteria (6 patients); had positive drug screen for illegal substances (4 patients); unwilling, unable, or inadvisable to discontinue prohibited medications during washout (3 patients). DB = double blind, ITT = intent to treat, OL = open label

The milnacipran program for JFM was conducted at 47 study sites in the United States. Patients who met the enrollment criteria entered the open-label period of the randomized withdrawal study and were treated with milnacipran (up to 100 mg/day in divided doses). Responders, as defined by stringent pre-defined criteria (described below), were then randomized to continue milnacipran or switch to placebo. Patients who were randomized and entered the double-blind withdrawal period, those who did not meet randomization criteria, and those who dropped out for reasons other than tolerability or adverse effects, were permitted to enter the open-label extension study (Fig. 1). Both studies were conducted in full compliance with the US Food and Drug Administration guidelines for good clinical practice and in accordance with Declaration of Helsinki principles. Approvals were obtained from all Institutional Review Boards prior to the start of the study. Written assent from each patient and written consent from his or her parent, legal guardian, or other legal representative were also obtained.

### Randomized withdrawal study

#### Eligibility criteria

Male or female outpatients, 13 to 17 years of age with a diagnosis of FM based on Yunus and Masi criteria [6] and/or the 1990 ACR criteria [7], were eligible to enter this study. Patients were also required to have an average pain score of 3 to 9 at screening and previous unsatisfactory response to a nonpharmacologic FM treatment. Pain scores were defined as the 1-week average of daily pain ratings on an 11-point scale that ranged from 0 (“no pain”) to 10 (“pain as bad as you can imagine”).

Patients were excluded from the study if they had a current severe psychiatric illness as determined by the Investigator or based on responses to the Mini International Neuropsychiatric Interview for Children and Adolescents (MINI-KID [34]). Patients with significant risk of suicidality were also excluded based on the Investigator’s judgment and review of the following: patient and guardian responses to the MINI-KID; suicidal ideation (levels 3, 4, or 5) or any suicidal behavior within the past 6 months, as reported using the electronic Columbia–Suicide Severity Rating Scale (C-SSRS) [35]. Other key exclusion criteria included: active liver disease or severe renal impairment; history of seizure disorder other than febrile seizures; any psychiatric or medical condition that could interfere with study conduct, confound the interpretation of study results, or endanger the patient’s well-being. Prohibited medications included any centrally acting pharmacotherapies commonly used to treat FM. Washout of these medications was required prior to entering the study. Nonpharmacologic therapies (e.g., physical therapy, acupuncture, chiropractic manipulation, CBT or other psychotherapy, biofeedback) were allowed provided that the treatments were initiated >30 days prior to screening and continued without major change during the studies. A more detailed list of eligibility criteria is provided in Additional file 1: Table S1.

#### Study conduct

Patients taking prohibited medications were washed out for up to 4 weeks before entering the 8-week open-label period of the randomized withdrawal study. During the first 4 weeks of this open-label period, milnacipran doses were escalated to 100 mg/day if tolerated. The dosing regimen was as follows: 12.5 mg on the first day; 12.5 mg twice daily (BID) for days 2 to 7; 25 mg BID for days 8 to 14; 37.5 mg BID for days 15 to 21; and 50 mg BID for days 22 to 28. Patients who could not tolerate the minimum dose of 50 mg/day were discontinued from the study. Those who tolerated at least the minimum dose then received 4 weeks of open-label milnacipran at the maximum tolerated stable dose (MTD; 50, 75, or 100 mg/day).

At the conclusion of the 8-week open-label treatment period, patients with ≥50 % pain improvement from baseline were considered eligible for double-blind randomization. Patients who met the ≥50 % pain improvement criterion were randomized (2:1) to milnacipran or placebo (i.e., continue treatment at the MTD or discontinue treatment, respectively). Patients with <50 % pain improvement after the open-label period were not randomized to the double-blind withdrawal period. However, these patients were permitted to enroll directly into the extension study. After completing or discontinuing prematurely from the randomized withdrawal study, patients who did not want to participate in the extension study were entered into a 1-week down-titration period.

### Extension study

Patients were allowed to enter the extension study if they had participated in the double-blind withdrawal study and tolerated the minimum dose of 50 mg/day. Premature discontinuation from the lead-in study (unless due to an adverse effect of the medication) was not a reason for exclusion. As described previously for the open-label period of the randomized withdrawal study, patients in the extension study underwent dose escalation for up to 4 weeks until they reached the MTD. They then continued to receive milnacipran at the MTD for up to 48 weeks. Those unable to tolerate the minimum dose of 50 mg/day were discontinued from the extension study and entered into a 1-week down-titration period.

### Outcome measures

A timeline of study assessments is presented in Additional file 1: Table S2. For the open-label period of the randomized withdrawal study, mean changes from baseline to end of treatment (i.e., last available assessment in the open-label period for each patient) were assessed in the following measures: 1-week average pain severity rating, calculated from daily pain ratings that were recorded by patients once-daily every morning using an interactive voice response and/or web response system; Patient Global Impression of Severity (PGIS), Pediatric Quality of Life Inventory-Teen Report (PedsQL [36]), and the Multidimensional Anxiety Scale for Children (MASC [37]). Although the Children’s Depression Inventory (CDI [38]) was primarily used as a safety assessment to monitor changes in depressive symptoms, mean changes in CDI total score are shown with outcome results in this report.

The PGIS consisted of a single question (“Considering all aspects of your illness, how do you evaluate the severity of your fibromyalgia?”) with scores ranging from 1 (“normal, not at all ill”) to 7 (“extremely ill”). The PedsQL, which has been validated in children and adolescents with FM [36], includes the following assessments: the 23-item Generic Core Scales, which covers physical, emotional, social, and school functioning; and the 18-item Multidimensional Fatigue Scale, which covers general fatigue, sleep/rest, and cognitive fatigue. Raw scores for the PedsQL were reversed and transformed to a 0–100 scale, with higher scores indicating better quality of life. The MASC is a 39-item assessment that evaluates physical symptoms, social anxiety, harm avoidance, and separation/panic anxiety. The CDI is a 27-item instrument that assesses the existence and severity of depressive symptoms in the following areas: negative mood, ineffectiveness, negative self-esteem, anhedonia, appetite, and interpersonal problems. Higher total scores indicate greater symptom severity for both the MASC (range, 0 to 117) and CDI (range, 0 to 54).

For the double-blind period of the randomized withdrawal study, the primary endpoint was time to loss of therapeutic response (LTR) from randomization to end of double-blind treatment. LTR was defined as any of the following events: worsening of JFM requiring alternate treatment; increase in pain score to >70 % of baseline; or withdrawal from the study for an adverse event (AE). In order to determine LTR, Investigators reviewed patients’ daily pain ratings at each study visit. They also assessed whether patients’ responses to the current treatment were sufficiently inadequate (based on worsening of JFM symptoms and/or lack of tolerability) to justify discontinuing the current treatment and implementing an alternate treatment; the type of alternate treatment was left to the Investigator’s discretion. Secondary endpoints were the mean score changes from randomization to end of double-blind treatment in pain, PGIS, PedsQL (Generic Core Scales, Multidimensional Fatigue Scale), and MASC.

Outcomes in the open-label extension study were evaluated based on mean score changes from baseline of the randomized withdrawal study to end of treatment (i.e., last available assessment for pain, PGIS, PedsQL, MASC, or CDI in each patient). The interactive web/voice response system was not used to collect pain data in the extension study; instead, the 11-point pain scale (range, 0–10) was administered at all study visits to assess each patient’s average pain over the past week.

### Safety measures

Safety and tolerability assessments included AE reporting, vital signs (sitting blood pressure, sitting heart rate, and body weight), electrocardiogram readings, and clinical laboratory tests. Changes in depressive and other psychiatric symptoms, as well as suicidal ideation and behavior, were monitored using the CDI and C-SSRS.

In the randomized withdrawal study, treatment-emergent AEs (TEAEs) for each period were defined as AEs that began or increased in severity after the first treatment dose of that period or within 30 days after the last dose of that period (in patients who discontinued or completed treatment). In the extension study, TEAEs were defined as AEs that started after the first dose of open-label treatment in the randomized withdrawal study or were present before this dose but increased in severity during the extension study. Newly emergent AEs (NEAEs) in the extension study were defined as AEs that occurred after the first dose of extension study treatment or increased in severity during the extension study.

Mean changes in vital signs, electrocardiograms, and laboratory tests, as well as the percentage of individual patients with potentially clinically significant (PCS) changes in these parameters, were analyzed for each study.

### Statistical analyses

In the randomized withdrawal study, the open-label safety and intent-to-treat (ITT) populations included screened patients who received ≥1 dose of open-label study medication. The double-blind safety and ITT populations included all randomized patients who took ≥1 dose of double-blind study medication. In the open-label extension study, the safety and ITT populations included screened patients who received ≥1 dose extension study medication.

The primary efficacy parameter in the initial study was time to first LTR. Based on the available literature for milnacipran in adult FM [22, 24, 25] and assuming LTR rates of 60 % for placebo and 35 % for milnacipran, it was estimated that 156 patients (placebo, 52; milnacipran, 104) would be needed to detect the above treatment difference with a power of 80 % or higher at the 2-sided 5 % significance level test. Assuming a completer/responder rate of approximately 50 %, it was estimated that 312 patients would need to participate in the open-label treatment period. The extension study was a follow-up to the randomized withdrawal study; therefore, sample size was not based on statistical considerations. However, due to unexpected low enrollment and early termination of the studies, all efficacy and safety outcomes were analyzed descriptively.

## Results

### Patients

Of 171 screened patients, 116 met the eligibility criteria and entered the open-label period of the randomized withdrawal study; all of these patients received ≥1 dose of open-label treatment and were included in the safety and ITT populations (Fig. 1). In this open-label population, 96 (82.8 %) patients were diagnosed with FM based on both the Yunus and Masi criteria and the 1990 ACR criteria; 19 (16.4 %) patients met only the Yunus and Masi criteria and 1 patient (0.9 %) met only the ACR criteria (Table 1). The majority of patients were female (84.5 % [98/116]) and white (86.2 % [100/116]), with a mean age of 15.6 years (Table 2). Patients commonly reported history of headache (34.5 %), migraine (22.4 %), insomnia (17.2 %), depressive symptoms (16.4 %), gastroesophageal reflux disease (14.7 %), back pain (12.1 %), and dysmenorrhea (12.1 %) (Table 1).

**Table 1:** Medical and psychiatric history in patients entering the open-label period of the randomized withdrawal study

	Patients
	*N* = 116
Fibromyalgia diagnosis, n (%)	
Yunus and Masi criteria only	19 (16.4)
1990 ACR criteria only	1 (0.9)
Both criteria	96 (82.8)
Psychiatric disorders or symptoms, n (%)^a^	
Insomnia	20 (17.2)
Depression	19 (16.4)
Anxiety	11 (9.5)
Attention deficit/hyperactivity disorder	8 (6.9)
Sleep disorder	5 (4.3)
Lifetime suicidality, n (%)	
Suicidal ideation	24 (20.7)
Suicidal behavior	4 (3.4)
Pain disorders or symptoms, n (%)^a^	
Headache	40 (34.5)
Migraine	26 (22.4)
Gastroesophageal reflux disease	17 (14.7)
Back pain	14 (12.1)
Dysmenorrhea	14 (12.1)
Irritable bowel syndrome	8 (6.9)
Arthralgia	7 (6.0)
Fatigue	6 (5.2)
Restless leg syndrome	5 (4.3)
Abdominal pain	4 (3.4)
Hypermobility syndrome	4 (3.4)
Abdominal pain upper	3 (2.6)
Myalgia^b^	3 (2.6)
Raynaud’s phenomenon	3 (2.6)
Temporomandibular joint syndrome	3 (2.6)
Other medical symptoms or conditions, n (%)^c^	
Asthma	27 (23.3)
Drug hypersensitivity	21 (18.1)
Seasonal allergy	19 (16.4)
Tonsillectomy	18 (15.5)

^a^Includes MedDRA preferred terms related to symptoms or disorders that have been associated with fibromyalgia. Additional terms of potential interest that were reported in 1 patient each: bipolar disorder, carpal tunnel syndrome, chronic fatigue syndrome, dysthymic disorder, juvenile arthritis, major depression, obsessive-compulsive disorder, osteoarthritis, osteochondrosis, panic attack, post-traumatic stress disorder, psychotic disorder, seasonal affective disorder, social phobia, spinal osteoarthritis, tendonitis, tic; scoliosis was reported in 5 patients

^b^Does not include symptoms that would be classified under the MedDRA preferred term of “fibromyalgia”

^c^Includes all other MedDRA preferred terms that were reported in ≥10 % of the open-label population

ACR = American College of Rheumatology

**Table 2:** Demographics and treatment dosages

	Randomized withdrawal study		Extension study
	Nonrandomized	Randomized	All patients
	*n* = 96	*n* = 20	*n* = 57
Age, years, mean (SD)	15.7 (1.3)	15.0 (1.6)	15.4 (1.4)
Female, n (%)	86 (89.6)	12 (60.0)	50 (87.7)
White, n (%)	86 (89.6)	14 (70.0)	51 (89.5)
BMI, kg/m^2^, mean (SD)	25.8 (6.2)	24.8 (5.3)	26.1 (6.3)
Maximum tolerated dose^a^			
50 mg/day	6 (6.3)	1 (7.1)^a^	3 (5.3)
75 mg/day	9 (9.4)	4 (28.6)^a^	8 (14.0)
100 mg/day	69 (71.9)	9 (64.3)^a^	46 (80.7)
No available MTD data	12 (12.5)	0^a^	0

^a^As reported in the milnacipran group only (*n* = 14)

BMI = body mass index, MTD = maximum tolerated dose, SD = standard deviation

MTD = maximum tolerated dose

All 20 patients who met the criteria for randomization received ≥1 dose of double-blind treatment and were included in the safety and ITT populations (Fig. 1). Two patients in the milnacipran group did not complete the double-blind randomized withdrawal period: 1 due to an AE, and 1 due to insufficient therapeutic response. A total of 57 patients (44 from the open-label population, 13 from the double-blind population) entered the extension study, all of whom were included in the safety and ITT populations. The extension study outcomes presented in this report are from patients who had available post-baseline assessments (i.e., *n* = 56 at Visit 2 [Week 4] of the extension study).

Based on available data, the majority of patients in both the randomized withdrawal study and the extension study had an MTD of 100 mg/day (Table 2). The mean treatment duration for the open-label period of the randomized withdrawal study was 52.3 days in nonrandomized patients and 56.8 days in patients who were subsequently randomized to double-blind treatment. The mean duration of double-blind treatment was 53.0 days. In the extension study, the mean duration of treatment was 143.6 days, with minimum and maximum durations of 8 and 370 days, respectively.

### Outcome measures

#### Randomized withdrawal study

At the end of open-label treatment with milnacipran in the randomized withdrawal study, mean improvements were found in pain, global disease severity, quality of life, and fatigue symptoms (Table 3). Patients with severe psychiatric comorbidities were excluded from the study, but mean changes on the MASC and CDI indicated no worsening of anxiety or depressive symptoms during this period. As expected, all mean score changes were greater in patients who met the pain criterion for randomization (i.e., ≥50 % decrease in pain severity from open-label baseline) than in patients who did not. A post hoc analysis of pain data from the nonrandomized population showed that although these patients did not meet the ≥50 % responder criterion after open-label milnacipran treatment, 27.7 % of them did experience a ≥30 % decrease in pain severity, which has been defined as a threshold of clinically relevant improvement in adult FM patients [39].

**Table 3:** Mean changes from baseline in the open-label period of the randomized withdrawal study

	Nonrandomized		Randomized	
	*n* = 96		*n* = 20	
Measures, mean (SD)	Baseline	Change^a^	Baseline	Change^a^
Pain, range 0-10	6.5 (1.4)	−1.1 (1.6)	6.1 (1.3)	−3.3 (1.0)
PGIS, range 1-7	4.0 (0.8)	−0.4 (1.0)	4.0 (0.8)	−1.0 (1.0)
PedsQL-Generic Core Scales, range 0-100	55.6 (14.8)	4.5 (13.0)	54.2 (11.4)	10.9 (10.9)
PedsQL-Multidimensional Fatigue Scale, range 0-100	46.1 (17.6)	5.8 (15.3)	41.3 (13.3)	13.3 (12.1)
MASC, range 0-117	45.1 (16.5)	−2.4 (12.2)	46.1 (13.4)	−5.4 (13.2)
CDI, range 0-54^b^	11.9 (7.1)	−1.2 (5.0)	11.1 (5.5)	−2.4 (4.9)

^a^Changes are based on end-of-treatment values. Negative changes represent mean improvements in pain, PGIS, MASC, and CDI scores; positive values represent mean improvements in PedsQL scores. For pain, data were based on electronic entries from Week 7 (i.e., the last week prior to randomization). For other measures, data were based on last available open-label assessments, including those from patients who prematurely discontinued the study

^b^Primarily used in this study as a safety assessment to monitor changes in depressive symptoms

CDI = Children’s Depression Inventory, MASC = Multidimensional Anxiety Scale, PGIS = Patient Global Impression of Severity, PedsQL = Pediatric Quality of Life Inventory-Teen Report, SD = standard deviation

Only 20 patients were eligible for randomization into the double-blind period. An LTR due to worsening of FM requiring alternative treatment was detected in 2 patients from the milnacipran group; time to LTR was 6 days in 1 patient, 8 days in the other. Slight worsening in PGIS, PedsQL, and MASC scores were found in both the placebo and milnacipran groups.

#### Extension study

**Fig. 2:**
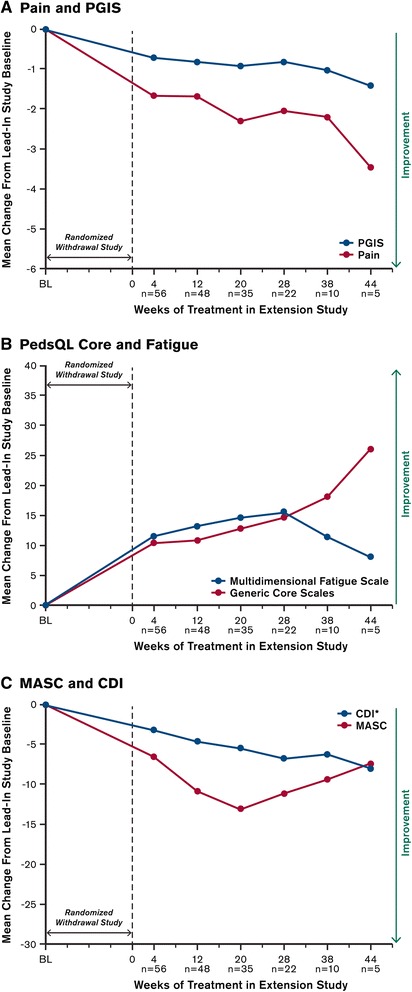
Mean changes from baseline in outcome measures during the extension study. Baseline was defined as pre-treatment values (i.e., patients’ scores prior to receiving the first dose of milnacipran in the open-label period of the randomized withdrawal study). For each visit in the extension study, baseline only includes pre-treatment values for those patients who completed that particular study visit. The n-values represent numbers of patients with valid assessments at baseline and at each extension study visit; graph only includes study visits that had >1 patient. *CDI was primarily used as a safety outcome. CDI = Children’s Depression Inventory, MASC = Multidimensional Anxiety Scale for Children, PedsQL = Pediatric Quality of Life Inventory, PGIS = Patient Global Impression of Severity

During the extension study, continued mean improvements from the lead-in study baseline in pain scores—and to a lesser extent, in the PedsQL Generic Core Scales, MASC, and CDI scores—were observed by Week 4 (i.e., end of dose escalation to MTD in the extension study) and at subsequent study visits (Fig. 2). A post hoc analysis of pain data from this study showed that at Week 20, the last time point at which >50 % of entering patients were still participating in the study (*n* = 35), 65.7 % of patients had ≥30 % pain improvement from the beginning of the prior randomized withdrawal study (Additional file 1: Figure S1). No mean worsening of depressive symptoms were detected in patients who participated in the extension study.

### Safety

#### Adverse events

No serious AEs were reported during the open-label period of the randomized withdrawal study. During the double-blind period, 1 patient randomized to milnacipran had a serious AE of suicidal ideation, along with a nonserious AE of anxiety, and was discontinued from the study for these reasons. One patient from the extension study, who had already discontinued milnacipran treatment due to nausea, experienced suicidal ideation 45 days after the last dose of study drug. This patient was not considered on-therapy at the time of suicidal ideation, and this event was judged by the Investigator as unrelated to treatment. Another patient in the extension study had a serious AE of cholecystitis and successfully underwent a cholecystectomy. Milnacipran was suspended for 11 days; treatment was resumed after the patient was discharged from the hospital. No deaths occurred during the randomized withdrawal study or the extension study.

Eight patients discontinued the open-label treatment period of the initial study due to an AE. Four of these patients reported nausea, which was the only AE that led to discontinuation in >1 patient. Aside from the patient who discontinued due to the serious AE of suicidal ideation (described above), no other patients discontinued the double-blind period due to an AE. TEAEs occurred more frequently during the open-label period (78.4 % [91/116]) than in the double-blind period (50.0 % [10/20]) (Table 4). TEAEs that occurred in >10 % of patients during the open-label period were nausea (32.8 %), vomiting (13.8 %), and headache (10.3 %).

**Table 4:** Treatment-emergent adverse events

	Randomized withdrawal study			Extension study
	Open-label	Double-blind		
	Milnacipran	Placebo	Milnacipran	Milnacipran
Patients, n (%)	*n* = 116	*n* = 6	*n* = 14	*N* = 57
Any TEAE^a^	91 (78.4)	4 (66.7)	6 (42.9)	42 (73.7)
Nausea	38 (32.8)	0	0	10 (17.5)
Vomiting	16 (13.8)	0	0	5 (8.8)
Headache	12 (10.3)	1 (16.7)	0	4 (7.0)
Dizziness	10 (8.6)	0	0	3 (5.3)
Fatigue	7 (6.0)	0	0	1 (1.8)
Hot flush	7 (6.0)	0	0	2 (3.5)
Tachycardia^b^	7 (6.0)	0	1 (7.1)	6 (10.5)
Decreased appetite	5 (4.3)	0	0	6 (10.5)
Hyperhidrosis	5 (4.3)	0	0	1 (1.8)
Insomnia	5 (4.3)	0	0	1 (1.8)
Upper respiratory tract infection	5 (4.3)	0	1 (7.1)	0
Urinary tract infection	5 (4.3)	0	0	5 (8.8)
Abdominal pain	4 (3.4)	0	0	4 (7.0)
Gastroenteritis	4 (3.4)	0	0	1 (1.8)
Heart rate increased^b^	4 (3.4)	0	0	4 (7.0)
Nasopharyngitis	4 (3.4)	0	0	2 (3.5)
Diarrhea	3 (2.6)	0	0	1 (1.8)
Dysmenorrhea	3 (2.6)	0	0	0
Irritability	3 (2.6)	0	0	0
Palpitations	3 (2.6)	0	0	1 (1.8)
Rash	3 (2.6)	0	0	0
Tremor	3 (2.6)	0	0	1 (1.8)

^a^Reported in ≥2 % of patients during the open-label period of the randomized withdrawal study; coded by MedDRA preferred term

^b^Tachycardia refers to an increase in heart rate that is greater than the age-corrected upper limit of normal. Heart rate increased refers to any increase, whether or not within the normal age-corrected range

TEAE = treatment-emergent adverse event

In the extension study, 10.5 % of patients discontinued due to an AE. The percentage of patients with any TEAE or NEAE was 73.7 % and 64.9 %, respectively. TEAEs that were reported in >10 % of patients in the extension study were nausea (17.5 %), tachycardia (10.5 %), and decreased appetite (10.5 %); these were considered to be NEAEs in 8.8 %, 3.5 %, and 3.5 % of patients, respectively (Additional file 1: Figure S2). In both studies, most patients had TEAEs that were categorized as either mild or moderate in severity (open-label period, 96.7 %; double-blind period, 83.3 % for milnacipran; extension, 92.9 %).

#### Vital signs

At the end of open-label treatment in the randomized withdrawal study, mean increases from baseline were found in sitting blood pressure (4 to 5 mm Hg) and sitting heart rate (10 bpm) (Table 5). At least once during this period, 6.9 % of the 116 patients met the PCS criteria for elevated systolic blood pressure (≥130 mm Hg with an increase from baseline of ≥20 mm Hg) and 9.5 % met the criteria for elevated diastolic blood pressure (≥80 mm Hg with increase ≥20 mm Hg); 9.5 % met the PCS criteria for elevated heart rate (≥110 bpm with increase of ≥30 bpm) (Additional file 1: Table S3). During the double-blind period, no PCS criteria were found in >1 patient in either treatment group.

**Table 5:** Mean changes in vital signs

	Randomized withdrawal study			Extension study
	Open-label period	Double-blind period		
Vital signs, mean (SD)	Milnacipran *n* = 116	Placebo *n* = 6	Milnacipran *n* = 14	Milnacipran *n* = 57
Systolic BP, mm Hg				
Baseline^a^	113.3 (9.7)	117.3 (7.9)	115.0 (7.7)	112.5 (8.7)
Change	4.1 (8.9)	−1.2 (6.8)	−1.4 (10.9)	2.5 (10.7)
Diastolic BP, mm Hg				
Baseline^a^	69.5 (7.8)	76.3 (9.3)	70.8 (10.4)	68.1 (8.7)
Change	5.2 (8.6)	−7.0 (8.3)	−0.1 (9.9)	4.2 (8.8)
Heart rate, bpm				
Baseline^a^	81.0 (11.1)	100.5 (17.9)	87.1 (17.5)	81.6 (10.8)
Change	10.0 (14.4)	−6.8 (16.0)	0.1 (18.6)	4.3 (11.1)
Body weight, kg				
Baseline^a^	69.4 (17.6)	71.9 (20.7)	64.7 (14.2)	68.9 (18.1)
Change	−0.8 (2.0)	1.9 (1.7)	−0.1 (1.9)	−0.5 (3.9)

^a^For the open-label period of the randomized withdrawal study and for the extension study, baseline was defined as the last available assessment before the first dose of open-label treatment in the randomized withdrawal study. For the double-blind period, baseline was defined as the last available assessment prior to the first dose of double-blind treatment

BP = blood pressure

Additional increases in sitting blood pressure and heart rate were not apparent with longer treatment duration. Among the 57 patients who entered the extension study and were included in safety analyses, mean increases from baseline of the prior randomized withdrawal study were 3 to 4 mm Hg for blood pressure and 4 bpm for heart rate (Table 5). During the open-label extension study, PCS criteria for elevated systolic and diastolic blood pressure were detected in 10.9 % and 12.7 % of patients, respectively; PCS criteria for elevated heart rate were detected in 12.7 % of patients (Additional file 1: Table S3).

Patients receiving milnacipran had slight (<1 kg) mean decreases in body weight after 8 weeks of open-label treatment in the randomized withdrawal study (mean −0.8 kg; range, −5.2 to +5.9 kg) and also in the extension study (mean, −0.5 kg; range, −9.6 to +7.7 kg) (Table 5). The percentage of patients meeting PCS criteria for weight change in the randomized withdrawal study or the extension study are reported in Additional file 1: Table S3.

#### Electrocardiograms

An increased heart rate (≥20 bpm) was found in 37.5 % of patients in the open-label period of the randomized withdrawal study and 33.3 % of patients in the extension study (Additional file 1: Table S3). A total of 14 milnacipran-treated patients had a QRS interval ≥100 msec: 6 during the open-label period of the randomized withdrawal study, 7 during the extension study, and 1 during both studies. As expected with increased heart rate, approximately 10 % of milnacipran-treated patients had a QTcB >450 msec in either study. However, no patient had an elevated QTcF of clinical significance.

#### Laboratory tests

For each laboratory test, 2 or fewer milnacipran-treated patients had a PCS value during either study (Additional file 1: Table S4).

## Discussion

Although it was important to evaluate the effects of an approved FM medication in adolescents, designing and conducting an appropriate clinical trial proved to be challenging. The responder-enriched, randomized withdrawal design used for the initial study was chosen in anticipation of the practical difficulties—and potentially ethical problem—of recruiting pediatric patients with a chronic pain condition into a more conventional placebo-controlled trial in which some participants would not receive active treatment. The milnacipran program allowed all eligible patients to receive at least 8 weeks of open-label milnacipran at their MTD. Decreases in dosing were also allowed to improve tolerability and retention. Moreover, patients were allowed to continue receiving long-term open-label treatment with milnacipran in the subsequent extension study even if they did not meet the pain response criterion (i.e., ≥50 % improvement from baseline) required for entry into the double-blind randomized withdrawal period. One drawback of the withdrawal study design was that a large number of patients was needed for the open-label period in order to evaluate efficacy (i.e., time to LTR in ≥50 % pain responders) with any statistical certainty. Although this was the same responder criterion used in randomized withdrawal studies of adult FM with both pregabalin [40] and milnacipran [41], a post hoc analysis of data from the milnacipran trial suggests that a less stringent cutoff (e.g., ≥30 % pain improvement) may have been adequate for evaluating loss of efficacy after treatment withdrawal [42].

It has been reported that >65 % of JFM patients have a current psychiatric disorder [10], and the exclusion of patients with severe psychiatric comorbidities may have limited enrollment in the milnacipran program. The general under-recognition of JFM may have also contributed to the difficulty of recruiting patients into the milnacipran program. Ongoing debates about how to define JFM can lead to delays in diagnosis and treatment for many patients [4], and some patients may not receive early medical attention for their condition. The diagnosis of JFM remains primarily symptom-based, and greater awareness among healthcare professionals is needed in order for patients to receive a more timely diagnosis. To that end, data collected from the 116 patients who entered the open-label period of the randomized withdrawal study provided useful information regarding the characteristics of adolescents who met the tender point and symptom criteria for FM, as assessed using the Yunus and Masi criteria [6], 1990 ACR criteria [7], or both. Specifically, the large majority of patients in this trial satisfied both sets of criteria; however, the Yunus and Masi criteria identified an important minority of patients (16.4 %) that were not identified by the ACR criteria. It is unclear whether this group might differ from patients satisfying the ACR criteria in response to treatment.

Similar to findings in adults with FM [43–46], the majority of participants in the milnacipran program were female, with mean baseline scores indicating substantial pain severity, fatigue symptoms, and reduced quality of life. Of the 116 patients who entered the open-label period of the randomized withdrawal study, 84.5 % were female. Mean baseline pain scores (>6 of 10) and PGIS scores (4 of 7) suggest that patients generally had moderate-to-severe symptoms prior to treatment. The mean baseline score for the PedsQL Generic Core Scales (55.4 for the total open-label population) was similar to the patient-reported score found by the Outcomes Measures in Rheumatology (OMERACT) Fibromyalgia Working Group in their validation study of this instrument (55.9) [36]. In the OMERACT study, the PedsQL Generic Core Scales was significantly lower in JFM patients than in pediatric cancer patients, rheumatology patients, and healthy controls (all p <0.001), indicating the negative impact of JFM on physical, psychosocial, and emotional functioning. The mean baseline score for the Multidimensional Fatigue Scale score was more severe in the milnacipran program (45.3) than in the OMERACT study (55.5), which emphasizes the importance of identifying and managing fatigue-related symptoms in patients with JFM.

Baseline mean MASC and CDI scores in the open-label population (Table 3) were somewhat higher (i.e., more severe) than normative scores found in a general adolescent population [47], although the CDI score was lower (i.e., less severe) than has been previously reported in JFM patients [3]. The percentage of patients in the milnacipran program with a history of depression (16.4 %) or anxiety (9.5 %) was also lower than has been previously reported in JFM [10]. These results are not surprising since patients with severe or unstable psychiatric disorders were excluded from the milnacipran program; similarly mild levels of depressive symptoms had been found in milnacipran studies that also excluded adult FM patients with severe psychiatric illness [22, 24, 25]. Other types of chronic conditions and pain-related symptoms seen in the milnacipran program (Table 1) were similar to those found in clinical studies of JFM [2, 3, 6] and in survey studies of adult FM [43, 48]. Interestingly, many of these symptoms (e.g., sleep disorder, irritable bowel syndrome, arthralgia, fatigue, hypermobility syndrome, temporomandibular joint syndrome) were also less prevalent (<10 %) than expected. Aside from the exclusion of patients with major psychiatric comorbidities, no compelling explanation can be provided for these results. On the other hand, it should be noted that >10 % of patients in this study did have a history of asthma (23.3 %), drug hypersensitivity (18.1 %), or seasonal allergy (16.4 %). It is difficult to speculate why these conditions were so common in the milnacipran JFM program, but research in adult FM patients suggests that neuroendocrine abnormalities—along with potential immunomodulatory effects of decreased serotonin levels on neuroendocrine systems—may play a role in the clinical overlap between chronic pain and inflammatory diseases [49, 50].

Low enrollment in the double-blind randomized withdrawal phase of the initial study, which was terminated, prevents interpretation of outcomes from that period. However, the open-label phases of the milnacipran program provide preliminary information about the potential effects of milnacipran in JFM. Mean changes in the largest patient sample (i.e., the 116 patients who entered the open-label period of the randomized study) and in the sample with the longest duration of treatment (i.e., the 56 patients who entered the extension study and had available outcome assessments) indicate that these patients, all of whom received open-label treatment with milnacipran, reported improvements in multiple symptom domains (Table 3 and Fig. 2). Although only 20 (17.2 %) of the 116 patients from the 8-week open-label period of the initial study met the ≥50 % pain responder criterion required for randomized withdrawal, 27.7 % reached a clinically meaningful threshold of ≥30 % pain improvement [39]. While no direct comparisons can be made due to marked differences in study design, results from the 12-week double-blind study of adult FM showed that 44.6 % of patients had ≥30 % pain improvement with milnacipran 100 mg/day, compared to 30.6 % in the placebo group (p < 0.001) [22].

Improvements in quality of life should also be noted since restored functioning is often considered to be as important as pain reduction in managing JFM [51]. No minimal clinically important differences for PedsQL have been established for JFM, but studies in other chronic childhood conditions suggest that a 4.5- to 6-point increase may reflect clinically relevant improvement [52, 53]. After open-label treatment in the randomized withdrawal study and during the extension the study, mean score changes in the PedsQL Generic Core Scales matched or exceeded these suggested thresholds. Improvements in this outcome measure, which encompasses various functional domains (i.e., physical, emotional, social, and school-related), along with the observed mean decreases in pain severity, suggest that patients with JFM may experience clinical benefits with milnacipran, particularly after several months of treatment.

Evaluating the safety and tolerability of milnacipran in JFM was an important aspect of this program, and it should be noted that dosing was based on tolerability. Overall, the tolerability profile in these patients (Table 4) was similar to the profile seen in adults with FM [31], with nausea, headache, vomiting, headache, and dizziness being the most commonly reported TEAEs after treatment was initiated in the open-label period of the randomized withdrawal study. The percentage of patients who discontinued this open-label period due to an AE (7 %) was lower than in placebo-controlled, fixed-dose studies of adult FM (23 % for the 100 mg/day dosage [31]); in both populations, however, nausea was the most common AE associated with discontinuation. The mean increases in blood pressure and heart rate found in the JFM population (Table 5) were also generally similar to the increases found in adults with FM [31]. These parameters were generally found to decrease after discontinuation of milnacipran in adults [54]. However, the long-term implications of milnacipran’s effects on blood pressure and heart rate are unknown.

Designing and conducting clinical trials in pediatric populations continues to be a challenge in many therapeutic areas [55]. In the milnacipran JFM program, the rationale for including an initial open-label treatment period was that it might induce more patients to enroll. However, this approach estimated that >300 patients would be needed to detect statistical significance in the double-blind withdrawal phase, which may have been too difficult a task given the general under-recognition of JFM [4]. In retrospect, it might have been easier to implement a more typical placebo-controlled, parallel-group design that would have required fewer patients. More flexible eligibility criteria might also have helped to increase enrollment—for example, allowing patients with stable mild-to-moderate major depression or patients requiring concomitant medications such as anticonvulsants for migraine. Within the study itself, a less onerous study visit schedule and simpler data collection techniques (e.g., more use of interactive web/voice response systems) might have helped to accommodate an adolescent population that may have been busy with competing interests such as school, social life, and extracurricular activities. Finally, although various tools were made available to Investigators to help recruit patients into the milnacipran program (e.g., website and mobile applications with study information and basic eligibility criteria, an online campaign to direct general web searches for JFM to the milnacipran program, press releases, support for print/radio/television advertising), continued efforts are needed to increase the general awareness of JFM. Such efforts could include reaching out to women with FM who might have at-risk children and reassuring them about clinical research in children, public service announcements about JFM, advertising with adult FM support groups, and outreach/education to pediatric rheumatologists.

The main limitations of the JFM milnacipran program have been discussed above, most notably with regards to study design, exclusion criteria, randomization criteria, and study termination due to low recruitment. Continued evaluation of the safety and efficacy of milnacipran and other pharmacotherapies in this patient population is warranted. Areas of potential interest include identifying predictors of pain response in JFM patients, exploring the relationship between pain relief and improvements in other symptom domains, and evaluating the effects of adjunctive nonpharmacologic therapies such as CBT and exercise.

## Conclusions

The milnacipran program showed that recruiting adolescent patients into a JFM treatment study can be a challenging proposition. Despite intensive recruitment efforts and the implementation of a randomized withdrawal paradigm that allowed patients to start by entering an open-label treatment period, only 171 patients from 47 study centers in the United States were screened for eligibility. Of these, 116 entered into the open-label period of the randomized withdrawal study, and only 20 patients were randomized for double-blind withdrawal. The ACR criteria for FM identified most patients with JFM in this program, but the Yunus and Masi criteria identified an additional 16.4 % of patients. Aside from exhibiting less severe depressive and anxiety symptoms due to eligibility criteria, the overall profile of these adolescent patients was not largely different from the profile of adult FM patients. Milnacipran was generally well tolerated, with more than two-thirds of the patients tolerating the maximum allowed dose of 100 mg/day and a side-effect profile similar to that seen in adults [31]. Results with open-label milnacipran treatment of JFM provided preliminary data that this medication may improve symptoms and function in some patients with JFM, but the pain responder rate was relatively low and controlled studies are needed to confirm these findings.

## Additional file

Additional file 1:
**Supplementary information and results.** This file includes the following: eligibility criteria at screening; timeline of assessments; potentially clinically significant changes in vital signs, electrocardiograms, and laboratory tests; and pain responders in the extension study, defined as patients with ≥30 % pain improvement from baseline of the randomized withdrawal study.

## Abbreviations

ACR: American College of Rheumatology
AE: Adverse event
BID: Twice daily
BMI: Body mass index
CBT: Cognitive behavioral therapy
CDI: Children’s Depression Inventory
C-SSRS: Columbia-suicide severity rating scale
FM: Fibromyalgia
ITT: Intent to treat
JFM: Juvenile fibromyalgia
LTR: Loss of therapeutic response
MASC: Multidimensional Anxiety Scale for Children
MINI-KID: Mini International Neuropsychiatric Interview for Children and Adolescents
MTD: Maximum tolerated dose
NEAE: Newly emergent adverse event
OMERACT: Outcome Measures in Rheumatology
PCS: Potentially clinically significant
PedsQL: Pediatric Quality of Life Inventory
PGIS: Patient Global Impression of Severity
TEAE: Treatment-emergent adverse event
VAS: Visual analog scale
