# Supplementary material for: Preliminary experience using milnacipran in patients with juvenile fibromyalgia: lessons from a clinical trial program
Source: Pediatr Rheumatol Online J. 2015 Jun 26;13:27. doi: 10.1186/s12969-015-0025-9 (PMC4480575; doi:10.1186/s12969-015-0025-9)
Supplement: Additional file 1: — Supplementary information and results. This file includes the following: eligibility criteria at screening; timeline of assessments; potentially clinically significant changes in vital signs, electrocardiograms, and laboratory tests; and pain responders in the extension study, defined as patients with ≥30 % pain improvement from baseline of the randomized withdrawal study. [file 12969_2015_25_MOESM1_ESM.pdf]

ADDITIONAL FILE 1

Table S1. Eligibility criteria

**Inclusion criteria**	• Male or female outpatients<br>• Ages 13 to 17 years, inclusive<br>• Primary diagnosis based on Yunus and Masi criteria and/or American College of Rheumatology criteria<br>• Average pain score ≥3 and ≤9 at screening (Visit 1)<br>• Previous unsatisfactory response to ≥1 nonpharmacologic fibromyalgia treatment<br>• Sufficiently stable home life for adequate safety monitoring<br>• Normal physical examination, electrocardiogram, and clinical laboratory tests<br>• Negative pregnancy test for female participants; adequate contraception for all participants<br>• Negative results for urine drug screen
**Exclusion criteria**	• Current severe psychiatric illness, based on Investigator's judgment or patient responses to the Mini International Neuropsychiatric Interview for Children and Adolescents (MINI-KID); applied to patients with current major depressive episode, major depressive disorder, suicidal thoughts or behavior, manic episode, hypomanic episode, bipolar disorder, psychotic disorder, mood disorder with psychotic features, or alcohol/substance abuse<br>• Significant risk of suicidality, based on Investigator's judgment or assessments at screening (MINI-KID, Columbia-Suicide Severity Rating Scale)<br>• Evidence of active liver disease (ie, aspartate aminotransferase, alanine aminotransferase, and/or alkaline phosphatase >1.5 times the upper limit of normal)<br>• Severe renal impairment (ie, creatinine clearance <30 mL/min)<br>• Alcohol or substance abuse within the previous year<br>• Current systemic infection (eg, human immunodeficiency virus, hepatitis), documented autoimmune disease, or history of seizure disorder other than febrile seizures<br>• Any psychiatric or medical condition that could interfere with study conduct, confound the interpretation of study results, or endanger patient wellbeing<br>• Any history or behavior that might prohibit compliance for the duration of the study, based on Investigator's judgment<br>• Experimental fibromyalgia treatment within 30 days or 5 half-lives (whichever is longer) prior to study entry<br>• History of intolerance or hypersensitivity to milnacipran

Table S1. Eligibility criteria (continued)

**Prohibited concomitant medications**	• All central nervous system (CNS)-active therapies commonly used to treat fibromyalgia<br>• Anorectics (eg, diethylpropion, sibutramine, phentermine, ephedrine, ma huang)<br>• Antidepressants (eg, MAO-A or -B inhibitors, tricyclic or tetracyclic antidepressants, SSRIs, SNRIs, NRIs)<br>• Antiepileptic agents (eg, phenytoin, topiramate, carbamazepine, levetiracetam, tiagabine, gabapentin, pregabalin)<br>• Opiates and related analgesics (eg, oxycodone, codeine, tramadol, narcotic patches)<br>• Dopamine agonists (eg, ropinirole, pramipexole)<br>• Stimulants (eg, dextroamphetamine mixed salts, methylphenidate, dextroamphetamine, modafinil, ephedrine, ma huang, atomoxetine)<br>• Muscle relaxants (eg, tizanidine, metaxalone, methocarbamol, carisoprodol)<br>• Miscellaneous excluded medications (eg, buspirone, sodium oxybate)
**Allowed concomitant medications**	• 5-hydroxytryptamine agonists (triptans) for migraines, with caution; Investigators were informed about serotonin syndrome or neuroleptic malignant syndrome–like reactions<br>• Combination agents consisting of butalbital, aspirin/acetaminophen, and caffeine for headaches; required discontinuation of these medications for at least 1 week prior to study visits<br>• Acetaminophen, aspirin, and other nonsteroidal anti-inflammatory drugs<br>• Sleep medications (eg, nonbenzodiazepine hypnotics per Investigator’s judgment, antihistamines, chloral hydrate)<br>• Epinephrine for severe allergic reaction; cautioned use of local anesthetics that contain epinephrine<br>• Nasal decongestants containing pseudoephedrine or herbal supplements containing stimulants, with cautioned use after consultation with Investigator<br>• Nonpharmacologic treatments (eg, physical therapy, acupuncture, chiropractic manipulation, massage therapy, cognitive behavioral therapy, psychological therapy, biofeedback) that were initiated >30 days prior to screening and continued without major change during the study

Table S2. Schedule of assessments

	Randomized Withdrawal Study											Extension Study								
	Screening/ Washout (1-4 Weeks)	Open-Label Period (8 Weeks)					Double-Blind Period (8 Weeks)				Down Titration[a] (1 Week)	Dose Escalation (4 Weeks)		Stable-Dose Period (48 Weeks)						Down Titration (1 Week)
**Study visit**	**SCR Wk -1 to -4**	**BL Wk 0**	**Wk 1[b]**	**Wk 2**	**Wk 3[b]**	**Wk 4**	**RA Wk 8**	**Wk 10**	**Wk 14**	**EOT/ Wk 16**	**Wk 17**	**BL Wk 0**	**Wk 4**	**Wk 12**	**Wk 20**	**Wk 28**	**Wk 36**	**Wk 44**	**EOT/ Wk 52**	**Wk 53**
Physical examination	X									X		X							X	
Vital signs	X	X		X		X	X	X	X	X	X	X	X	X	X	X	X	X	X	X
Clinical laboratory tests	X	X		X		X	X		X	X		X	X	X		X			X	
Electrocardiogram		X				X	X	X		X		X		X		X			X	
Adverse events	X	X	X	X	X	X	X	X	X	X	X	X	X	X	X	X	X	X	X	X
Pain rating scale	X	X	X	X	X	X	X	X	X	X		X	X	X	X	X	X	X	X	
Pain rating scale (daily compliance)[c]		X		X		X	X	X	X											
Alternative FM treatment assessment[d]								X	X	X										
Patient Global Impression of Severity	X	X		X		X	X	X	X	X		X	X	X	X	X	X	X	X	
Pediatric Quality of Life Inventory – Teen Report		X					X			X		X	X	X	X	X	X	X	X	
Multidimensional Anxiety Scale for Children		X					X			X		X	X	X	X	X	X	X	X	
Children's Depression Inventory		X					X			X		X	X	X	X	X	X	X	X	
Columbia-Suicide Severity Rating Scale (electronic version)	X	X		X		X	X	X	X	X	X	X	X	X	X	X	X	X	X	X
Mini International Neuropsychiatric Interview for Children and Adolescents	X																			

[a] In patients who received milnacipran ≥50 mg/day and did not continue into the extension study; [b] telephone visit; [c] study center personnel monitored patient compliance with entry of daily pain ratings via an interactive web or voice response system during the randomized withdrawal study; [d] patients were also allowed to contact study center personnel if an alternative treatment was required, with a study visit scheduled as needed.

BL=baseline; EOT=end of treatment (last study visit in patients who discontinued for any reason); FM=fibromyalgia; RA=randomization visit; SCR=screening visit.

Table S3. Potentially clinically significant vital signs and electrocardiograms

	Randomized Withdrawal Study			
	Open-Label Period	Double-Blind Period		Extension Study
PCS criteria, n/N (%)[a]	Milnacipran	Placebo	Milnacipran	Milnacipran
Vital signs				
SBP, mm Hg				
≥130 + ≥20 increase	8/116 (6.9)	0	1/14 (7.1)	6/55 (10.9)
≤90 + ≥20 decrease	0	0	0	0
≥140	5/116 (4.3)	0	0	3/55 (5.5)
DBP, mm Hg				
≥80 + ≥20 increase	11/116 (9.5)	1/6 (16.7)	1/14 (7.1)	7/55 (12.7)
≤20 + ≥20 decrease	0	0	0	0
≥90	10/116 (8.6)	0	1/14 (7.1)	7/55 (12.7)
SBP and DBP, mm Hg				
≥140 and ≥90	4/116 (3.4)	0	0	2/55 (3.6)
Heart rate, bpm				
≥110 + ≥30 increase	11/116 (9.5)	0	0	7/55 (12.7)
≤55 + ≥30 decrease	0	0	0	0
Weight, kg				
≥7% increase	1/116 (0.9)	0	1/14 (7.1)	7/55 (12.7)
≥7% decrease	3/116 (2.6)	0	0	13/55 (23.6)
Electrocardiograms				
Heart rate ≥120 bpm	0	0	0	0
Heart rate ≤50 bpm	1/112 (0.9)	0	0	1/51 (2.0)
Heart rate increase ≥20 bpm	42/112 (37.5)	0	1/14 (7.1)	17/51 (33.3)
QRS ≥100 msec	7/103 (6.8)	2/6 (33.3)	0	8/49 (16.3)
PR >200 msec	0	0	0	0
QTcB >450 msec	10/110 (9.1)	1/6 (16.7)	1/14 (7.1)	6/49 (12.2)
QTcF >450 msec	0	0	0	0

[a] For increases and decreases, baseline was defined as follows: for the open-label period and extension study, last available assessment before the first dose of open-label treatment in the randomized withdrawal study; for the double-blind period, last available assessment before the first dose of double-blind treatment in the randomized withdrawal study.

DBP=diastolic blood pressure, n=number of patients meeting PCS criteria, N=number of patients with ≥1 post-baseline assessment, PCS=potentially clinically significant, SBP=systolic blood pressure.

Table S4. Potentially clinically significant laboratory values

	Randomized Withdrawal Study			
	Open-Label Period	Double-Blind Period		Extension Study
PCS criteria, n/N (%)[a]	Milnacipran	Placebo	Milnacipran	Milnacipran
Eosinophils, percent >2 ∗ ULN	2/91 (2.2)	0	0	0
Lymphocytes, percent <0.6 ∗ LLN	2/92 (2.2)	0	0	2/52 (3.8)
White blood cells, 10^9/L <0.7 ∗ LLN	2/115 (1.7)	0	0	2/55 (3.6)
ALT (SGPT), U/L >3 ∗ ULN	0	0	0	2/55 (3.6)
AST (SGOT), U/L >3 ∗ ULN	0	0	0	2/55 (3.6)

[a] Table includes laboratory values that were met by ≥2% of patients during the open-label period of the randomized withdrawal study or by ≥2% of patients during the extension study. Baseline was defined as the last available assessment before first dose open-label treatment in the randomized withdrawal study.

ALT (SGPT)=alanine aminotransferase (serum glutamic-pyruvic transaminase), AST (SGOT)=aspartate aminotransferase (serum glutamic oxaloacetic transaminase); LLN=lower limit of normal range; n=number of patients meeting PCS criteria, N=number of patients with a non-PCS baseline and ≥1 post-baseline assessment, PCS=potentially clinically significant, ULN=upper limit of normal range.

Figure S1. Pain responders in the open-label extension study

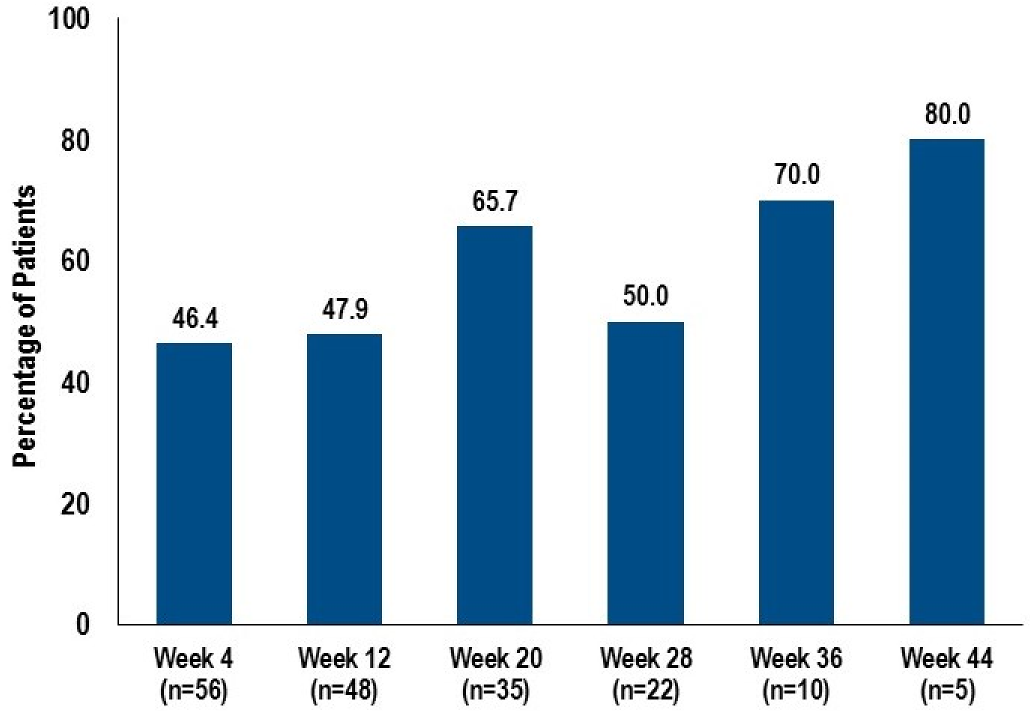

Figure shows the percentage of patients at each study visit who had ≥30% pain improvement from baseline, defined as the weekly average of daily pain ratings that were taken before start of open-label treatment in the prior randomized withdrawal study. The n-values represent numbers of patients at each extension study visit with available pain assessments; graph only includes study visits that had >1 patient.

Figure S2. Treatment-emergent adverse events (TEAEs) and newly emergent adverse events (NEAEs) in the open-label extension study

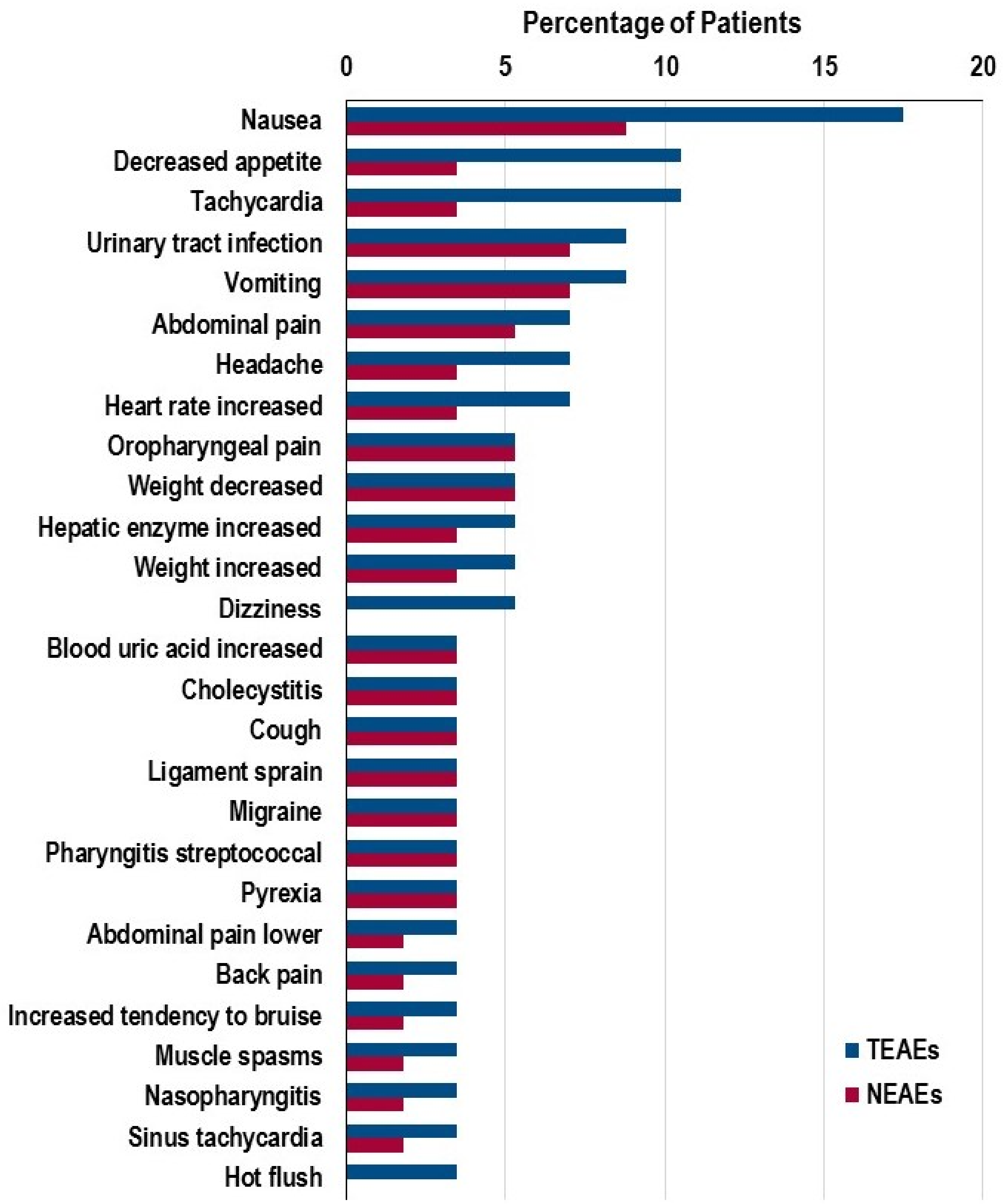

Figure shows all TEAEs that were reported in ≥2% of patients in the safety population.
